# A novel stereographic semi-circular distribution and enhanced analysis of the posterior corneal curvature of eye

**DOI:** 10.1016/j.heliyon.2024.e40487

**Published:** 2024-11-19

**Authors:** Shakila Bashir, Bushra Masood, Aamir Sanaullah, Laila A. Al-Essa

**Affiliations:** a Department of Statistics, Forman Christian College (A Chartered University), Lahore, Pakistan; b Department of Statistics, COMSATS University Islamabad, Lahore Campus, Pakistan; c Department of Mathematical Sciences, College of Science, Princess Nourah bint Abdulrahman University, P.O.Box 84428, Riyadh, 11671, Saudi Arabia

**Keywords:** Semi-circular, Marshall-Olkin, Extended burr-XII, Trigonometric moments, Stereographic projection, *l*-axial

## Abstract

Circular distributions within the (−π,π) radians range describe two-dimensional directions by mapping points onto a unit circle. These distributions are vital in diverse fields such as medicine, ecology, and environmental studies, where measurements are expressed in terms of angles. However, when these distributions involve measuring angles within the (0,π) radians range, they constitute axial or semi-circular data instead of circular data. This research seeks to introduce the semi-circular Marshall-Olkin extended Burr-XII distribution tailored for semi-circle datasets. Objectives encompass presenting its fundamental characteristics and applications. The inverse stereographic projection technique is applied for its development, deriving characteristics like trigonometric moments, mode, hazard function, and survival function. Five estimation techniques assess the distribution's parameters. Monte Carlo simulations evaluate parameter estimation methods for different sample sizes. Modeling the semi-circular Marshall-Olkin extended Burr-XII distribution with real-life semi-circle data of posterior corneal curvature of eye demonstrates its adaptability. Comparisons with existing distributions affirm its effectiveness. Extending to the *l*-axial model produces the Stereographic-*l*-axial Marshall-Olkin extended Burr-XII distribution, offering a distinct probability density function (pdf). This transformation gives rise to specific scenarios and new models. The proposed semi-circular Marshall-Olkin extended Burr-XII distribution proves adept at handling real-world semi-circular data. The extension to the *l*-axial model and subsequent transformations introduces innovative models, demonstrated by superior compatibility in both circular and semi-circular datasets.

## 1 Introduction

The statistical field of circular data analysis is between spherical and linear data analysis. In the biological, medicinal, and meteorological disciplines, observations are measured using circular or directed data. Circular distributions are important in many fields, including modeling cross-bedding data, researching pale currents, detecting wind directions, assessing temporal patterns in crime occurrence, and analyzing Mother's Day ceremonies.

When the total probability is shown on a unit circle, the distribution is known as a circular distribution. It is a way to assign probability to various directions since every point on the unit circle corresponds to a direction. One can interpret the range of the circular random variable θ, expressed in radians, as either (0,2π) or (-π, π). A set of points can be represented as points on the circumference of a circle with a unit radius in two-dimensional data. These kinds of data are represented by models called circular distributions, Shahsanaei et al. [1]. Both discrete and continuous circular distributions are distinguished by the probability they give to a finite number of directions and an infinite number of directions, respectively. Several methods may be used to create different efficient circular models using the real line or plane's current probability densities. Several well-known existing linear or circular probability distributions can be utilized to generate circular models using these methods/techniques. According to Mardia and Jupp [2], there are several sources of circular data. The two primary methods align with the two primary circular measuring devices, the clock and the compass. The compass is typically used to measure observations such as wind directions and bird migration paths. Similar types of data are derived from protractor or spirit level measurements. The arrival times of patients at a hospital's casualty unit, observed on a 24-h clock, are an example of typical observations recorded by the clock. Similar types of data appear at the proper times of year (or month) for relevant occurrences. It is clear from previous studies that these methods do not focus on semi-circular or axial observations. According to Alldredge et al. [3], axial data analysis has numerous applications, including examining fracture orientations in rock formations, studying grain orientations in polycrystalline materials, and analyzing wind direction patterns in meteorology. Circular observations are sometimes expressed as modulo π. Few illustrations are: (i)- The optical axis of a crystal (instead of a direction) or the long axis of sedimentary particles., (ii)- Core samples orientations, (iii)- A sea turtle emerges from the ocean to find a dry place to nest., (iv)- Semi-circular models are necessary to track the body parts of an aircraft loss situation given the angles of beginning direction and departure, (v)- An ornithologist observing the departure of sea birds form the shear face of a cliff need only record angles from within (0, π) radians. Hence, in these cases, full circular models are not important and as noted by Ahn and Kim [4] and, Yedlapalli et al. [5]. They highlighted this problem and framed a methodology for deriving distributions which are suitable for modelling these types of data.

A semi-circular distribution is defined as.

i. $\int_{0}^{\pi }f\left(\theta \right)d\theta =1,\;0<\theta <\pi \text{.}$
ii. f(θ)≥0
iii. f(θ)=f(θ+π).

It is discussed in Subrahmanyam [6] that when an inverse stereographic projection is used on linear models with support (0, ∞), the resulting distributions are Stereographic semi-circular models that are automatically mapped onto (0, π).

Breitenberger [7] introduced circular and spherical analogs of the normal distribution. Jammalamadaka and Kozubowski [8] discussed about how the classical exponential and Laplace distributions from the real line were wrapped onto the circle to make circular distributions, Cremers and Klugkist [9] Ali and Sana presented a circular data analysis tutorial in cognitive psychology that uses R and includes examples, Joshi and Jose [10] developed a Circular distribution named as wrapped Lindley distribution, Girija [11] developed a discrete wrapped Cauchy model, Al-Khazaleh and Alkhazaleh [12] investigated wrapping of Quasi Lindley distribution, Tahir et al. [13] used doubly censored data to study Bayesian estimation of the mixture of Burr Type-XII distributions. Maruthan and Bava [14] proposed circular Generalized Logistic Distributions, three circular distributions were introduced through the utilization of inverse stereographic projection names as CGL-I distribution, two parameters CGL-I distribution and CGL-II distribution.

Less research has been done on semi-circular distributions. Abuzaid [15] developed a half-circular Burr-XII distribution to simulate the curvature of the posterior cornea, Rambli [16] proposed a half circular transformed gamma distribution using the inverse stereographic projection technique. Iftikhar et al. [17] developed half circular modified Burr−III (hc-MB-III) distribution and applications with some parameters estimation methods, Oleiwi et al. [18] developed transformed semicircular exponentiated Weibull distribution, Abe et al. [19] introduced family of symmetric unimodal distributions utilizing a modified stereographic projection, Abid [20] presented a stereographic generalized inverse Weibull distribution, Yedlapalli et al. [21] developed transmuted semicircular distribution and applied it on geology. Although the above-mentioned literature is the semi-circular development on some of the well-known continuous distributions, particularly burr family exhibits more flexibility on semi-circular data sets, see, (Abuzaid [15], and Iftikhar et al. [17]). Yedlapalli et al. [22] developed Semicircular Arc Tan-Exponential Type distribution. In this study a more general form of the burr distribution named Marshall-Olkin extended (MOE) Burr-XIII is considered due to the more pertinency of the burr family in the under-study area.

In the domain of circular data analysis, this study addresses a critical research gap by introducing the stereographic semi-circular MOE (StSC-MOE) Burr-XII distribution, specifically tailored for semi-circle data sets prevalent in fields like meteorology, biology, and medical sciences. The study aims to comprehensively explore the StSC-MOE Burr-XII distribution's fundamental characteristics, including its mode, hazard function, survival function, and trigonometric moments, while also evaluating five estimation techniques for parameter estimation. Through monte Carlo simulations and real-life applications, the study demonstrates the distribution's practical utility in handling semi-circular data, exemplified by posterior corneal curvature measurements. Furthermore, the research extends the distribution to the *l*-axial model, broadening its applicability to arcs of varying lengths. Remarkably, the study's innovative approach involves applying transformations to the SC-MOE Burr-XII distribution, resulting in the development of Stereographic Circular MOE (StC-MOE) Burr-XII and Stereographic Circular (StC) Burr-XII distributions, offering novel alternatives for circular data analysis. These contributions collectively advance the field of circular data analysis by introducing a versatile distribution, providing insights into estimation techniques, and offering innovative circular models, ultimately enhancing our ability to model and analyze semi-circular data effectively.

In many real-life scenarios, the data or the understudied variable often doesn't conform to simple measurement. Instead, the data may take on directional, circular, or semi-circular forms. For instance, consider the posterior corneal curvature of the eye, wind direction, the release of homing pigeons, and the movement of sea stars. In these cases, the data is not only directional but also takes on a semi-circular form. Therefore, there is a need to develop more directional probability distributions. Although some work has been done on a few probability distributions, it is understood that every probability distribution rarely fits on every situation. Moreover, a very little work has been done on semi-circular probability distributions as compared to circular distributions. However, when the real-life data is strictly into the radians [0,π), there is a need to model the semi-circular probability distributions. To address this deficiency, the proposed StSC-MOE-Burr-XII distribution is developed particularly for modeling the semi-circular data sets. The proposed distribution is applied on a semi-circular data set, namely the posterior corneal curvature of the eye, and demonstrate flexibility over competitive distributions. Later the proposed StSC-MOE-Burr-XII model is transformed into *l*-axial MOE-Burr-XII distribution which contains special cases and are applicable to both circular and semi-circular data sets according to the specific conditions.

This article presents the research's findings in the following way: in section 2, StSC-MOE Burr-XII has been developed, graphical representation and some basic properties along with reliability measures are derived, and in section 3 trigonometric moments and some of characteristics of SC-MOE Burr-XII distribution are presented, *l*-axial MOE Burr-XII distribution is introduced with special cases and graphs in section 4, parameters estimation is discussed in section 5 and in section 6, 7 simulation study and applications has been shown.

## 2 Material and methodology: proposed stereographic semi-circular MOE Burr-XII distribution

Burr [23] introduced the Burr distribution, originally referred to as the Burr Type XII distribution, within a set of twelve continuous distribution types forming the Burr system for analyzing lifetime data. Characterized by a decreasing hazard rate function resembling an upside-down bathtub shape, this distribution holds significant utility in survival analyses, particularly within the medical field for modeling survival times. Rodriguez [24] provided a comprehensive guide to the Burr-XII distributions, which are extensively employed among the Burr distribution system. The inverse of the Burr-XII density is referred to as the Burr-III density, characterized by two parameters. These distributions exhibit diverse shapes and find applications across various disciplines such as medical sciences, chemical engineering, business, quality control, and reliability studies. Notably, the Burr-XII distribution garners considerable attention from researchers due to its broad implications. Serving as a framework encompassing Weibull and logistic distributions as sub-models, it is commonly utilized for modeling lifetime data and phenomena with varying failure rates, capable of accommodating a wide range of empirical data. Through manipulation of different parameter values, the Burr-XII distribution can effectively capture a spectrum of skewness and kurtosis, thus facilitating the simulation of diverse data types in domains including finance, hydrology, and reliability analysis. Its versatility extends to simulating data pertaining to household income, crop prices, insurance risk, flood levels, and failure data.

Al-Saiari et al. [25] introduced a MOE Burr-XII distribution with the cumulative distribution function (cdf) given by

(1) $$F\left(x;\alpha ,\beta ,\gamma \right)=\frac{1-{\left(1+x^{\beta }\right)}^{-\gamma }}{1-\left(1-\alpha \right){\left(1+x^{\beta }\right)}^{-\gamma }},\;x,\;\alpha ,\;\beta ,\;\text{and}\;\gamma \;>\;0\text{,}$$

and the probability density function (pdf) corresponding to Eq. (1) given by

(2) $$f\left(x;\alpha ,\beta ,\gamma \right)=\frac{{\alpha \beta \gamma x^{\beta -1}\left(1+x^{\beta }\right)}^{-\gamma -1}}{{\left[1-\left(1-\alpha \right){\left(1+x^{\beta }\right)}^{-\gamma }\right]}^{2}},\;x,\alpha ,\beta ,\;\text{and}\;\gamma \;>\;0\text{.}$$

A stereographic projection arises from the mobius transformation is stated as

(3) $$m\left(\theta \right)=x=u+v\;\frac{\sin\left(\theta \right)}{1+\cos\left(\theta \right)}=u+v\;\tan\left(\frac{\theta }{2}\right)\text{.}$$

A one-to-one mapping that defines inverse stereographic projection is stated as

(4) $$m\left(\theta \right)=x=\left\{ \begin{array}{c} u+v\;\tan\left(\frac{\theta }{2}\right),\;x\in \left(-\infty ,\infty \right),\;\theta \in \left(-\pi ,\pi \right),\;u\in R,\;v>0,\\ u+v\;\tan\left(\frac{\theta }{2}\right),\;x\in \left(0,\infty \right),\;\theta \in \left(0,\pi \right),\;u\in R,\;v>0. \end{array}\right.$$

Now, considering, (v,u)=(1,0) in Eq. (2), we get a modified inverse stereographic projection as $2\;\tan^{-1}\left(x\right)=\theta$, where, θ∈(0,π). A StSC-MOE Burr-XII distribution is obtained by incorporating the inverse stereographic projection into Eq. (2). The pdf of StSC-MOE Burr-XII distribution is derived by following,

(5) $$g\left(\theta \right)=\left|m^{\prime }\left(\theta \right)\right|f\left[m\left(\theta \right)\right]\text{.}$$

Considering $m\left(\theta \right)=\tan\frac{\theta }{2}$, we have

(6) $$\left|m^{\prime }\left(\theta \right)\right|=\left|\frac{1}{2}\sec^{2}\left(\theta /2\right)\right|\;\text{and},f\left[m\left(\theta \right)\right]=\frac{{\alpha \beta \gamma {\left(\tan\frac{\theta }{2}\right)}^{\beta -1}\left[1+{\left(\tan\left(\frac{\theta }{2}\right)\right)}^{\beta }\right]}^{-\gamma -1}}{{\left[1-\left(1-\alpha \right){\left\{1+{\left(\tan\left(\frac{\theta }{2}\right)\right)}^{\beta }\right\}}^{-\gamma }\right]}^{2}}\;\text{.}$$

Now, incorporating Eq. (6) into Eq. (5), the pdf of StSC-MOE Burr-XII distribution is obtained and given by

(7) $$g\left(\theta \right)=\frac{\alpha \beta \gamma }{2}\sec^{2}\left(\frac{\theta }{2}\right){\left(\tan\frac{\theta }{2}\right)}^{\beta -1}\frac{{\left[1+{\left\{\tan\left(\frac{\theta }{2}\right)\right\}}^{\beta }\right]}^{-\gamma -1}}{{\left[1-\left(1-\alpha \right){\left\{1+{\left(\tan\left(\frac{\theta }{2}\right)\right)}^{\beta }\right\}}^{-\gamma }\right]}^{2}},\text{for}\;0<\;\theta <\;\pi \text{,}$$

where, α is a scale parameter, and β & γ are the shape parameters.

Similarly, the cdf corresponding to Eq. (7) is given as

$$G\left(\theta \right)=P\left(\omega \leq \theta \right)=P\left(2\;\tan^{-1}\;X\leq \theta \right)\text{,}$$

$$G\left(\theta \right)=P\left(X\leq 2\;\tan^{-1}\left(\frac{\theta }{2}\right)\right)\text{,}$$

$$G\left(\theta \right)=\int_{0}^{\tan\left(\frac{\theta }{2}\right)}F\left(x\right)dx\text{,}$$

(8) $$G\left(\theta \right)=\frac{1-{\left[1+{\left\{\tan\left(\frac{\theta }{2}\right)\right\}}^{\beta }\right]}^{-\gamma }}{1-\left(1-\alpha \right){\left[1+{\left\{\tan\left(\frac{\theta }{2}\right)\right\}}^{\beta }\right]}^{-\gamma }},\;0<\;\theta <\;\pi \text{.}$$

**Fig. 1:**
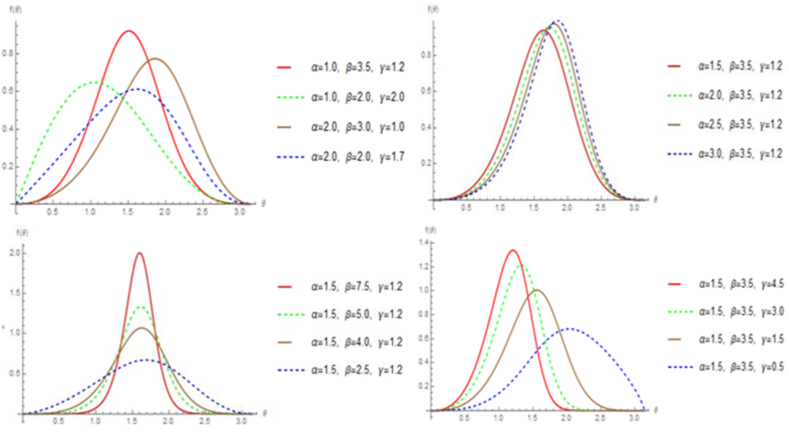
Linear presentation of StSC-MOE Burr-XII distribution.

**Fig. 2:**
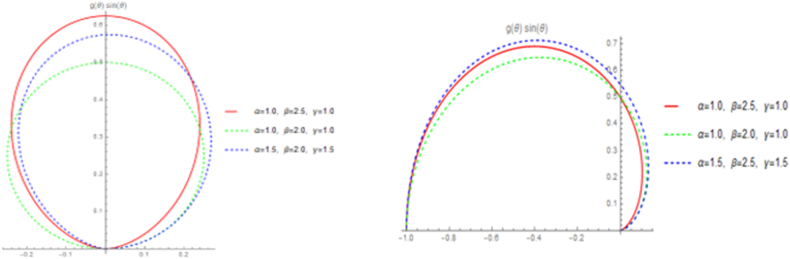
Circular presentation of StSC-MOE Burr-XII distribution with its cdf

Fig. 1 illustrates that the StSC-MOE Burr-XII distribution, with its pdf given by Eq. (7), shows a variety of shapes including symmetric, left-skewed, right-skewed, mesokurtic, and platykurtic shapes. This flexibility reflects its adaptability to different kind of data. Similarly, Fig. 2 displays different circular representations of the cdf of StSC-MOE Burr-XII, as given by Eq. (8).

The survival function of StSC-MOE Burr-XII distribution is given by

$$S\left(\theta \right)=\alpha {\left[1+{\left\{\tan\left(\frac{\theta }{2}\right)\right\}}^{\beta }\right]}^{-\gamma }\text{,}$$

and, the hazard function of the StSC-MOE Burr-XII distribution is given by

(9) $$h\left(\theta \right)=\frac{\beta \gamma }{2}\sec^{2}\left(\frac{\theta }{2}\right){\left(\tan\frac{\theta }{2}\right)}^{\beta -1}\frac{{\left[1+{\left\{\tan\left(\frac{\theta }{2}\right)\right\}}^{\beta }\right]}^{-1}}{{\left[1-\left(1-\alpha \right){\left\{1+{\left(\tan\left(\frac{\theta }{2}\right)\right)}^{\beta }\right\}}^{-\gamma }\right]}^{2}}\text{.}$$

**Fig. 3:**
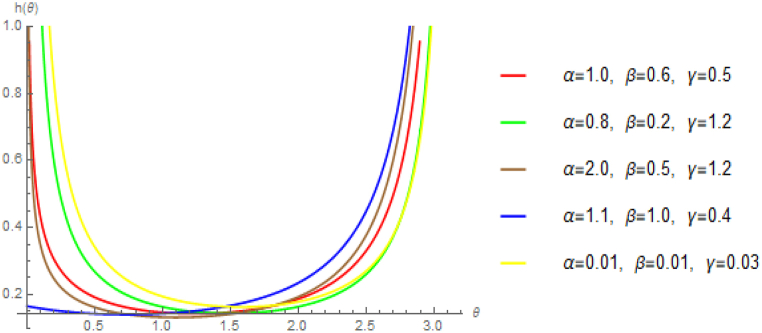
Hazard rate plot of semi-circular MOE Burr-XII distribution.

Fig. 3 demonstrates that the hazard rate of StSC MOE Burr-XII, given by Eq. (7), displays a bathtub shape, demonstrating the usefulness of the proposed distribution. A bathtub-shaped hazard function is particularly desirable in probability distributions, as it showcases the distribution's flexibility and applicability for variety of datasets. The availability of a range of lifetime distributions with a bathtub-shaped hazard function holds great importance, especially in analyzing the lifetimes of mechanical, electrical, and electronic products.

The reverse hazard rate of StSC-MOE Burr-XII distribution is given by

$$r_{h}\left(\theta \right)=\frac{\alpha \beta \gamma }{2}\sec^{2}\left(\frac{\theta }{2}\right){\left(\tan\frac{\theta }{2}\right)}^{\beta -1}\frac{{\left[1+{\left\{\tan\left(\frac{\theta }{2}\right)\right\}}^{\beta }\right]}^{-\gamma -1}}{\left[1-{\left\{1+{\left(\tan\left(\frac{\theta }{2}\right)\right)}^{\beta }\right\}}^{-\gamma }\right]\left[1-\left(1-\alpha \right){\left\{1+{\left(\tan\left(\frac{\theta }{2}\right)\right)}^{\beta }\right\}}^{-\gamma }\right]}\text{.}$$

The quantile function (QF) of the StSC-MOE Burr-XII distribution is given by

(10) θq=2tan−1[q(1−α)−1q−1]1βγ.

The median and Inter Quartile Range for the StSC-MOE Burr-XII distribution can be calculated as Median = $y_{0.5}$ and IQR = $y_{0.75}-y_{0.25}$.

Mode is obtained as the log of the probability distribution function of the proposed model,

Mode=ln[g(θ)],

$$Mode=\ln\left[\frac{\alpha \beta \gamma }{2}\sec^{2}\left(\frac{\theta }{2}\right){\left(\tan\frac{\theta }{2}\right)}^{\beta -1}\frac{{\left[1+{\left\{\tan\left(\frac{\theta }{2}\right)\right\}}^{\beta }\right]}^{-\gamma -1}}{{\left[1-\left(1-\alpha \right){\left\{1+{\left(\tan\left(\frac{\theta }{2}\right)\right)}^{\beta }\right\}}^{-\gamma }\right]}^{2}}\right]\text{.}$$

**Special case:** When α = 1, the StSC-MOE Burr-XII distribution in Eq. (7) is transformed into the semi-circular Burr-XII (StSC-Burr-XII) distribution given by

(11) $$g\left(\theta \right)=\frac{\beta \gamma }{2}\sec^{2}\left(\frac{\theta }{2}\right){\left(\tan\frac{\theta }{2}\right)}^{\beta -1}{\left[1+{\left\{\tan\left(\frac{\theta }{2}\right)\right\}}^{\beta }\right]}^{-\gamma -1},\;0<\;\theta <\;\pi \text{,}$$

and, the cdf, and hazard function corresponding to Eq. (11) are given respectively by

(12) $$G\left(\theta \right)=1-{\left[1+{\left\{\tan\left(\frac{\theta }{2}\right)\right\}}^{\beta }\right]}^{-\gamma },\;0<\;\theta <\;\pi \text{,}$$

and,

(13) $$h\left(\theta \right)=\frac{\beta \gamma }{2}\sec^{2}\left(\frac{\theta }{2}\right){\left(\tan\frac{\theta }{2}\right)}^{\beta -1}{\left[1+{\left\{\tan\left(\frac{\theta }{2}\right)\right\}}^{\beta }\right]}^{-1}\text{.}$$

## 3 Characteristic function and properties related to trigonometric moments for the StSC-MOE Burr-XII distribution

The characteristic function of the StSC-MOE Burr-XII distribution is defined as

$$\varphi_{q}=\int_{-\infty }^{\infty }e^{iqx}dF\left(x\right)\text{,}$$

or

(14) $$\varphi_{q}=\frac{\alpha \beta \gamma }{2}\int_{0}^{\pi }{e^{iq\theta }\;\sec}^{2}\left(\frac{\theta }{2}\right){\left(\tan\frac{\theta }{2}\right)}^{\beta -1}\frac{{\left[1+{\left\{\tan\left(\frac{\theta }{2}\right)\right\}}^{\beta }\right]}^{-\gamma -1}}{{\left[1-\left(1-\alpha \right){\left\{1+{\left(\tan\left(\frac{\theta }{2}\right)\right)}^{\beta }\right\}}^{-\gamma }\right]}^{2}}d\theta \text{.}$$

Alternatively, Eq. (14) is considered to be

$$\varphi_{q}=\alpha_{q}+i\beta_{q}\text{,}$$

or,

$$\varphi_{q}=\int_{-\infty }^{\infty }\;\mathrm{Cos}\left(qx\right)\;dF\left(x\right)+i\int_{-\infty }^{\infty }\;\mathrm{Cos}\left(qx\right)\;dF\left(x\right)\text{.}$$

Consider,

$$\alpha_{q}=E\left[\mathrm{Cos}\left(q\theta \right)\right]=\int_{0}^{\pi }\;\mathrm{Cos}\left(q\theta \right)g\left(\theta \right)\;d\theta \text{,}$$

and

$$\beta_{q}=E\left[\mathrm{Sin}\left(q\theta \right)\right]=\int_{0}^{\pi }\;\mathrm{Sin}\left(q\theta \right)g\left(\theta \right)\;d\theta \text{.}$$

Consequently,

(15) $$\begin{array}{c} \alpha_{q}=E\left[\mathrm{Cos}\left(q\theta \right)\right]=E\left[\mathrm{Cos}\left(-q\theta \right)\right]=\alpha_{-q}\text{,}\\ {-\beta }_{q}=E\left[\mathrm{Sin}\left(-q\theta \right)\right]=-E\left[\mathrm{Sin}\left(q\theta \right)\right]=\beta_{-q}\text{,}\\ \left|\alpha_{q}\right|,\left|\beta_{q}\right|\leq 1\text{.} \end{array}\}$$

The trigonometric moments as given by Eq. (15) are the qth-order sine and cosine moments of the θ, which are utilized to find the characteristics of the proposed distribution. The real parts of the qth-order trigonometric moments are given by

(16) $$\begin{array}{c} \alpha_{q}=\frac{\alpha \beta \gamma }{2}\int_{0}^{\pi }\;\mathrm{Cos}\left(q\theta \right)\sec^{2}\left(\frac{\theta }{2}\right){\left(\tan\frac{\theta }{2}\right)}^{\beta -1}\frac{{\left[1+{\left\{\tan\left(\frac{\theta }{2}\right)\right\}}^{\beta }\right]}^{-\gamma -1}}{{\left[1-\left(1-\alpha \right){\left\{1+{\left(\tan\left(\frac{\theta }{2}\right)\right)}^{\beta }\right\}}^{-\gamma }\right]}^{2}}\;d\theta \text{,}\\ and\\ \beta_{q}=\frac{\alpha \beta \gamma }{2}\int_{0}^{\pi }\;\mathrm{Sin}\left(q\theta \right)\sec^{2}\left(\frac{\theta }{2}\right){\left(\tan\frac{\theta }{2}\right)}^{\beta -1}\frac{{\left[1+{\left\{\tan\left(\frac{\theta }{2}\right)\right\}}^{\beta }\right]}^{-\gamma -1}}{{\left[1-\left(1-\alpha \right){\left\{1+{\left(\tan\left(\frac{\theta }{2}\right)\right)}^{\beta }\right\}}^{-\gamma }\right]}^{2}}\;d\theta \text{.} \end{array}\}$$

Considering *q* = 1 in Eq. (16), the 1st-order sine and cosine moments are given by

(17) $$\begin{array}{c} \alpha_{1}=\frac{\alpha \beta \gamma }{2}\int_{0}^{\pi }\;\mathrm{Cos}\left(\theta \right)\sec^{2}\left(\frac{\theta }{2}\right){\left(\tan\frac{\theta }{2}\right)}^{\beta -1}\frac{{\left[1+{\left\{\tan\left(\frac{\theta }{2}\right)\right\}}^{\beta }\right]}^{-\gamma -1}}{{\left[1-\left(1-\alpha \right){\left\{1+{\left(\tan\left(\frac{\theta }{2}\right)\right)}^{\beta }\right\}}^{-\gamma }\right]}^{2}}\;d\theta \text{,}\\ and\\ \beta_{1}=\frac{\alpha \beta \gamma }{2}\int_{0}^{\pi }\;\mathrm{Sin}\left(\theta \right)\sec^{2}\left(\frac{\theta }{2}\right){\left(\tan\frac{\theta }{2}\right)}^{\beta -1}\frac{{\left[1+{\left\{\tan\left(\frac{\theta }{2}\right)\right\}}^{\beta }\right]}^{-\gamma -1}}{{\left[1-\left(1-\alpha \right){\left\{1+{\left(\tan\left(\frac{\theta }{2}\right)\right)}^{\beta }\right\}}^{-\gamma }\right]}^{2}}\;d\theta \text{.} \end{array}\}$$

The central trigonometric moments of the StSC-MOE Burr-XII distribution are defined as

(18) $$\begin{array}{c} \alpha_{q}^{*}=E\left[\mathrm{Cos}\left(q\left(\theta -\mu \right)\right)\right]=\int_{0}^{\pi }\;\mathrm{Cos}\left(q\left(\theta -\mu \right)\right)g\left(\theta \right)\;d\theta \text{,}\\ and\\ \beta_{q}^{*}=E\left[\mathrm{Sin}\left(q\left(\theta -\mu \right)\right)\right]=\int_{0}^{\pi }\;\mathrm{Sin}\left(q\left(\theta -\mu \right)\right)g\left(\theta \right)\;d\theta \text{.} \end{array}\}$$

So, the 1st-order and 2nd-order central trigonometric moments are obtained considering *q* = 1 & 2 and, are given respectively by

(19) $$\&\begin{array}{c} \alpha_{1}^{*}=\frac{\alpha \beta \gamma }{2}\int_{0}^{\pi }\;\mathrm{Cos}\left(\left(\theta -\mu \right)\right)\sec^{2}\left(\frac{\theta }{2}\right){\left(\tan\frac{\theta }{2}\right)}^{\beta -1}\frac{{\left[1+{\left\{\tan\left(\frac{\theta }{2}\right)\right\}}^{\beta }\right]}^{-\gamma -1}}{{\left[1-\left(1-\alpha \right){\left\{1+{\left(\tan\left(\frac{\theta }{2}\right)\right)}^{\beta }\right\}}^{-\gamma }\right]}^{2}}\;d\theta \text{,}\\ \beta_{1}^{*}=\frac{\alpha \beta \gamma }{2}\int_{0}^{\pi }\;\mathrm{Sin}\left(\left(\theta -\mu \right)\right)\sec^{2}\left(\frac{\theta }{2}\right){\left(\tan\frac{\theta }{2}\right)}^{\beta -1}\frac{{\left[1+{\left\{\tan\left(\frac{\theta }{2}\right)\right\}}^{\beta }\right]}^{-\gamma -1}}{{\left[1-\left(1-\alpha \right){\left\{1+{\left(\tan\left(\frac{\theta }{2}\right)\right)}^{\beta }\right\}}^{-\gamma }\right]}^{2}}\;d\theta \text{.} \end{array}\}$$

and

(20) $$\&\begin{array}{c} \alpha_{2}^{*}=\frac{\alpha \beta \gamma }{2}\int_{0}^{\pi }\;\mathrm{Cos}\;2\left(\left(\theta -\mu \right)\right)\sec^{2}\left(\frac{\theta }{2}\right){\left(\tan\frac{\theta }{2}\right)}^{\beta -1}\frac{{\left[1+{\left\{\tan\left(\frac{\theta }{2}\right)\right\}}^{\beta }\right]}^{-\gamma -1}}{{\left[1-\left(1-\alpha \right){\left\{1+{\left(\tan\left(\frac{\theta }{2}\right)\right)}^{\beta }\right\}}^{-\gamma }\right]}^{2}}\;d\theta \text{,}\\ \beta_{2}^{*}=\frac{\alpha \beta \gamma }{2}\int_{0}^{\pi }\;\mathrm{Sin}\;2\left(\left(\theta -\mu \right)\right)\sec^{2}\left(\frac{\theta }{2}\right){\left(\tan\frac{\theta }{2}\right)}^{\beta -1}\frac{{\left[1+{\left\{\tan\left(\frac{\theta }{2}\right)\right\}}^{\beta }\right]}^{-\gamma -1}}{{\left[1-\left(1-\alpha \right){\left\{1+{\left(\tan\left(\frac{\theta }{2}\right)\right)}^{\beta }\right\}}^{-\gamma }\right]}^{2}}d\theta \text{.} \end{array}\}$$

Sever important results are derived for the proposed StSC-MOE-Burr-XII distribution and are given as follows, The mean direction:

(21) $$\mu =\tan^{-1}\left(\frac{\beta_{1}}{\alpha_{1}}\right)\text{.}$$

The mean resultant length (MRL):

(22) $$\rho =\sqrt{\alpha_{1}^{2}+\beta_{1}^{2}}\text{.}$$

The variance for the StSC-MOE Burr-XII distribution:

(23) $$v=1-\rho =1-\sqrt{\alpha_{1}^{2}+\beta_{1}^{2}},0<v<1\text{.}$$

The standard deviation for the proposed distribution:

(24) $$\sigma =\sqrt{-\log\left(\alpha_{1}^{2}+\beta_{1}^{2}\right)}\text{.}$$

The skewness and kurtosis:

(25) γ1=β2∗(1−ρ)32=β2(ρ2−2β12)−2α1α2β1ρ2(1−ρ)23.

and

(26) $$\gamma_{2}=\frac{\alpha_{2}^{*}-\rho^{4}}{{\left(1-\rho \right)}^{2}}\text{.}$$

The numerical values for these characteristics are computed following the expression given by Eq.s (17–26), and the results are presented in Table 1.

**Table 1:** Characteristics of StSC MOE Burr-XII distribution.

		α=2,andβ=2					β=2andγ=2				α=2andγ=2			
Parameter Values		γ=1	γ=2	γ=3	γ=4	γ=5	α=1	α=2	α=4	α=5	β=1	β=2	β=4	β=1
Mean Direction μ		−1.2809	1.4033	1.1730	1.0285	0.9271	1.1694	1.4033	−1.5073	−1.4356	1.3029	1.4033	1.4726	1.4896
Trigonometric Moments	$\alpha_{1}$	−0.2274	0.1416	0.3426	0.4679	0.5533	0.3333	0.1416	−0.0544	−0.1161	0.1777	0.1416	0.0929	0.0782
	$\alpha_{2}$	−0.2711	−0.4760	−0.4168	−0.3052	−0.1922	−0.3333	−0.4760	−0.5200	−0.5121	−0.0177	−0.4760	−0.7938	−0.8557
	$\beta_{1}$	0.7623	0.8375	0.8153	0.7766	0.7373	0.7854	0.8375	0.8550	0.8533	0.6472	0.8375	0.9430	0.9612
	$\beta_{2}$	−0.2616	0.1582	0.4337	0.5961	0.6922	0.3927	0.1582	−0.1094	−0.1950	0.1038	0.1582	0.1479	0.1331
Resultant Length ρ		0.7955	0.8494	0.8843	0.9066	0.9218	0.8532	0.8494	0.8568	0.8611	0.6711	0.8494	0.9476	0.9643
Variance u		0.2045	0.1506	0.1157	0.0934	0.0782	0.1468	0.1506	0.1432	0.1389	0.3289	0.1506	0.0524	0.0357
Central Trigonometric Moments	$\alpha_{1}^{*}$	−0.7955	0.8494	0.8843	0.9066	0.9218	0.8532	0.8494	−0.8568	−0.8611	0.6711	0.8494	0.9476	0.9643
	$\alpha_{2}^{*}$	0.3701	0.5016	0.6015	0.6696	0.7183	0.5140	0.5016	0.5297	0.5456	0.0682	0.5016	0.8074	0.8660
	$\beta_{1}^{*}$	0.0000	0.0000	0.0000	0.0000	0.0000	0.0000	0.0000	0.0000	0.0000	0.0000	0.0000	0.0000	0.0000
	$\beta_{2}^{*}$	0.0703	0.0071	0.4337	−0.0087	−0.0090	−0.0331	0.0071	0.0426	0.0511	−0.0802	0.0071	0.0098	0.0070
Skewness $\gamma_{1}$		0.7601	0.1217	−0.1484	−0.3066	−0.4128	−0.5879	0.1217	0.7852	0.9875	−0.4252	0.1217	0.8201	1.0394
Kurtosis $\gamma_{2}$		−0.7254	−0.8355	−0.7592	−0.6858	−0.6262	−0.7376	−0.8355	−0.4452	−0.2228	−1.2448	−0.8355	0.4128	0.9145
Standard deviation σ		0.6765	0.5714	0.4958	0.4428	0.4035	0.5635	0.5714	0.5560	0.5468	0.8931	0.5714	0.3282	0.2695

From Table 1, several observations are illustrated, such as.

• **When*α*and*β*are fixed, but*γ*increases****:**
  i. Mean direction is firstly increasing and then decreasing,
  ii. Variance is decreasing,
  iii. Skewness is positive skewed to negative skewed,
  iv. Kurtosis is less than 3.
• **When*γ*and*β*are fixed, but*α*increases:**
  i. Mean direction is firstly increasing and then decreasing.
  ii. Variance is decreasing.
  iii. Skewness is negative skewed to positive skewed.
  iv. Kurtosis is less than 3.
• **When*γ*and*α are fixed*, but*β*increases:**
  i. Mean direction is firstly increasing,
  ii. Variance is decreasing,
  iii. Skewness is negative skewed to positive skewed,
  iv. Kurtosis is less than 3.

## 4 Stereographic *l*-axial (Extemporaneous) MOE Burr-XII distribution

In this section, the StSC-MOE Burr-XII distribution is used to obtain *l*-axial MOE Burr-XII (la-MOE Burr-XII) distribution. la-MOE Burr-XII distribution is presented with its special cases and graphical presentations.

For continuous case, $g:[0,\frac{2\pi }{l})\to R,l\;\epsilon \;N$ is the pdf of a l-axial distribution, if g has the subsequent basic characteristics:

i- g(θ)≥0,∀θ
ii- $\int_{0}^{\frac{2\pi }{l}}g\left(\theta \right)d\theta =1$
iii- $g\left(\theta \right)=g\left(\theta +\frac{2k\pi }{l}\right),k\;\epsilon \;Z,l\;\epsilon \;N\text{.}$

The model proposed in Eq. (7) has been expanded to include the *l*-axial model, which is relevant to arcs of any length, such as 2πl where l belongs to the set of integers (l∈N). Extending the StSC-MOE Burr-XII distribution allows us to derive the Stereographic *la*-MOE Burr-XII distribution, which proves to be both feasible and valuable in various statistical analyses.

(27) $$g\left(\theta^{*}\right)=\frac{\alpha \beta \gamma l}{2}\sec^{2}\left(\frac{{l\theta }^{*}}{4}\right){\left[\tan\left(\frac{{l\theta }^{*}}{4}\right)\right]}^{\beta -1}\frac{{\left[1+{\left\{\tan\left(\frac{{l\theta }^{*}}{4}\right)\right\}}^{\beta }\right]}^{-\gamma -1}}{{\left[1-\left(1-\alpha \right){\left\{1+{\left(\tan\left(\frac{{l\theta }^{*}}{4}\right)\right)}^{\beta }\right\}}^{-\gamma }\right]}^{2}}\text{,}$$

where, $0<\;\theta^{*}<\;\frac{2\pi }{l}$, and, $\theta^{*}=\frac{2\pi }{l}$.

In specific cases, by setting the value of l as 1 or 2 in Eq. (27), we obtain new models. These models are denoted as four special cases, and are presented below.

**Table:** 

Case-I: for l=1	$g\left(\theta^{*}\right)=\frac{\alpha \beta \gamma }{4}\sec^{2}\left(\frac{\theta^{*}}{4}\right){\left[\tan\left(\frac{\theta^{*}}{4}\right)\right]}^{\beta -1}\frac{{\left[1+{\left\{\tan\left(\frac{\theta^{*}}{4}\right)\right\}}^{\beta }\right]}^{-\gamma -1}}{{\left[1-\left(1-\alpha \right){\left\{1+{\left(tan\left(\frac{\theta^{*}}{4}\right)\right)}^{\beta }\right\}}^{-\gamma }\right]}^{2}},0<\theta^{*}<2\pi$ circular MOE Burr-XII (C-MOE Burr-XII) distribution **(new model).**
Case-II: l=2	$g\left(\theta^{*}\right)=\frac{\alpha \beta \gamma }{2}\sec^{2}\left(\frac{\theta^{*}}{2}\right){\left[\tan\left(\frac{\theta^{*}}{2}\right)\right]}^{\beta -1}\frac{{\left[1+{\left\{\tan\left(\frac{\theta^{*}}{2}\right)\right\}}^{\beta }\right]}^{-\gamma -1}}{{\left[1-\left(1-\alpha \right){\left\{1+{\left(\tan\left(\frac{\theta^{*}}{2}\right)\right)}^{\beta }\right\}}^{-\gamma }\right]}^{2}},\;0<\theta^{*}<\pi$ the StSC-MOE Burr-XII distribution (same as proposed model).
Case-III: l=1,&α=1	$g\left(\theta^{*}\right)=\frac{\beta \gamma }{4}\sec^{2}\left(\frac{\theta^{*}}{4}\right){\left(\tan\frac{\theta^{*}}{4}\right)}^{\beta -1}{\left[1+{\left\{\tan\left(\frac{\theta^{*}}{4}\right)\right\}}^{\beta }\right]}^{-\gamma -1},\;0<\;\theta <\;2\pi$ circular Burr-XII distribution **(new model).**
Case-IV: l=2,&α=1	$g\left(\theta^{*}\right)=\frac{\beta \gamma }{2}\sec^{2}\left(\frac{\theta^{*}}{2}\right){\left(\tan\frac{\theta^{*}}{2}\right)}^{\beta -1}{\left[1+{\left\{\tan\left(\frac{\theta^{*}}{2}\right)\right\}}^{\beta }\right]}^{-\gamma -1},\;0<\theta^{*}<\pi$ StSC-Burr-XII distribution.

From above mentioned cases, Case-I represents the C-MOE Burr-XII distribution, designed for circular datasets. Case-II corresponds to the StSC-MOE Burr-XII distribution, identical to the proposed distribution. Case-III entails the circular Burr-XII distribution, while Case-IV involves the StSC-Burr-XII distribution. Density plots are provided for the two circular model cases, which are novel additions to the study.

## 5 Parameter estimation method

The parameters of the StSC-MOE Burr-XII distribution are estimated in this section using five different estimation techniques. These approaches include Maximum Likelihood Estimation (MLE) Cramér-von Mises (CVM), Ordinary least squares (OLS), Weighted least squares (WLS) and Anderson-Darling (AD) methods.

### 5.1 Maximum likelihood estimation

The most popular approach is maximum likelihood estimation used amongst all due to its elegant properties. Thus, applying the likelihood function corresponding to Eq. (7) is given by,

$$L\left(\vartheta \right)={\left(a\beta \lambda \right)}^{n}\sum_{i=1}^{n}\sec^{2}\left(\frac{\theta_{i}}{2}\right)\sum_{i=1}^{n}{\left(\tan\frac{\theta_{i}}{2}\right)}^{\beta -1}\sum_{i=1}^{n}\frac{{\left[1+{\left\{\tan\left(\frac{\theta_{i}}{2}\right)\right\}}^{\beta }\right]}^{-\gamma -1}}{{\left[1-\left(1-\alpha \right){\left\{1+{\left(\tan\left(\frac{\theta_{i}}{2}\right)\right)}^{\beta }\right\}}^{-\gamma }\right]}^{2}}\text{.}$$

The log-likelihood (LL) function l = l(ϑ) for a single observation θ is as given below

(28) $$l\left(\vartheta \right)=nlog\alpha +nlog\beta +nlog\gamma -nlog2+2\sum_{i=1}^{n}\log\left[\sec^{2}\left(\frac{\theta_{i}}{2}\right)\right]+\left(\beta -1\right)\sum_{i=1}^{n}\log\left[\tan\left(\frac{\theta_{i}}{2}\right)\right]-\left(\gamma +1\right)\sum_{i=1}^{n}\log\left[1+{\left\{\tan\left(\frac{\theta_{i}}{2}\right)\right\}}^{\beta }\right]-2\sum_{i=1}^{n}\log\left[1-\left(1-\alpha \right){\left\{1+{\left(\tan\left(\frac{\theta_{i}}{2}\right)\right)}^{\beta }\right\}}^{-\gamma }\right]\text{.}$$

The partial derivates of Eq. (28) with respective to each of the three parameters α, β, and γ, are obtained, and given as follows, respectively,

(29) $$\frac{dl}{d\alpha }=\frac{n}{\alpha }-2\sum_{i=1}^{n}\frac{{\left\{1+{\left(\tan\left(\frac{\theta }{2}\right)\right)}^{\beta }\right\}}^{-\gamma }}{1-\left(1-\alpha \right){\left\{1+{\left(\tan\left(\frac{\theta }{2}\right)\right)}^{\beta }\right\}}^{-\gamma }}\text{,}$$

(30) $$\frac{dl\left(\vartheta \right)}{d\beta }=\frac{n}{\beta }-\sum_{i=1}^{n}\frac{\log\left[\tan\left(\frac{\theta }{2}\right)\right]\left[\gamma {\left\{\tan\left(\frac{\theta }{2}\right)\right\}}^{\beta }+\gamma {\left\{\tan\left(\frac{\theta }{2}\right)\right\}}^{\beta }{\left\{1+{\left(\tan\left(\frac{\theta }{2}\right)\right)}^{\beta }\right\}}^{-\gamma }-{\left\{1+{\left(\tan\left(\frac{\theta }{2}\right)\right)}^{\beta }\right\}}^{-\gamma }-\alpha \gamma {\left\{\tan\left(\frac{\theta }{2}\right)\right\}}^{\beta }-\alpha +1\right]}{\left[{\left\{1+{\left(\tan\left(\frac{\theta }{2}\right)\right)}^{\beta }\right\}}^{-\gamma }+\alpha -1\right]\left[1+{\left(\tan\left(\frac{\theta }{2}\right)\right)}^{\beta }\right]}\text{,}$$

and

(31) $$\frac{dl\left(\vartheta \right)}{d\gamma }=\frac{n}{\gamma }-2\sum_{i=1}^{n}\frac{\log\left(1-\alpha \right){\left\{1+{\left(\tan\left(\frac{\theta }{2}\right)\right)}^{\beta }\right\}}^{-\gamma }}{{\left\{1+{\left(\tan\left(\frac{\theta }{2}\right)\right)}^{\beta }\right\}}^{-\gamma }\left[1-\left(1-\alpha \right){\left\{1+{\left(\tan\left(\frac{\theta }{2}\right)\right)}^{\beta }\right\}}^{-\gamma }\right]}-\sum_{i=1}^{n}\log\left[1+{\left(\tan\left(\frac{\theta }{2}\right)\right)}^{\beta }\right].$$

Since no closed form exists for the three equations (see Eqs. (29), (30), (31)), the parameter estimates can be obtained by numerically solving the non-linear system of equations ${{\left(\frac{{dl}_{n}^{*}}{d\alpha },\frac{{dl}_{n}^{*}}{d\lambda }\right)\;}^{T}=\left(0,0\right)}^{T}$.

### 5.2 Ordinary and weighted least squares estimation

Let $\theta_{1},\theta_{2},\ldots ,\theta_{n}$ is the random sample from a distribution with cumulative distribution function G(θ) and suppose that $\theta_{i},\;i=1,2,...,n$ denotes the order statistics. For a size *n*,

$$E\left[G\left(\theta_{\left(i\right)}\right)\right]=\frac{i}{\left(n+1\right)}\text{.}$$

The OLS estimator parameters α, β, and γ are estimated by minimizing the expression given by

(32) $$Q\left(\alpha ,\beta ,\gamma \right)=\sum_{i=1}^{n}{\left[G\left(\theta_{\left(i:n\right)}|\alpha ,\beta ,\gamma \right)-\frac{i}{\left(n+1\right)}\right]}^{2}\text{.}$$

Alternatively, Eq. (32) takes the form corresponding to Eq. (7), and is given by

(33) $$Q\left(\alpha ,\beta ,\lambda \right)=\sum_{i=1}^{n}{\left[\frac{1-{\left[1+{\left\{\tan\left(\frac{\theta }{2}\right)\right\}}^{\beta }\right]}^{-\gamma }}{1-\left(1-\alpha \right){\left[1+{\left\{\tan\left(\frac{\theta }{2}\right)\right\}}^{\beta }\right]}^{-\gamma }}-\frac{i}{\left(n+1\right)}\right]}^{2}\text{.}$$

The OLS estimates of the parameters α, β, and γ are obtained by taking the partial derivative of Equation (33) with respect to each parameter. After some mathematical simplifications, the OLS estimates are given as follows

$$\sum_{i=1}^{n}\left[\frac{1-{\left[1+{\left\{\tan\left(\frac{\theta }{2}\right)\right\}}^{\beta }\right]}^{-\gamma }}{1-\left(1-\alpha \right){\left[1+{\left\{\tan\left(\frac{\theta }{2}\right)\right\}}^{\beta }\right]}^{-\gamma }}-\frac{i}{\left(n+1\right)}\right]\Delta_{s}\left(y_{\left(i\right)}|\alpha ,\beta ,\gamma \right)=0,\;s=1,2,3$$

where,

$$\Delta_{1}\left(y_{\left(i\right)}|\alpha ,\beta ,\gamma \right)=\frac{1-{\left[1+{\left\{\tan\left(\frac{\theta }{2}\right)\right\}}^{\beta }\right]}^{-\gamma }}{{\left[{\left\{1+{\left(\tan\left(\frac{\theta }{2}\right)\right)}^{\beta }\right\}}^{-\gamma }+\alpha -1\right]}^{2}}\text{,}$$

$$\Delta_{2}\left(y_{\left(i\right)}|\alpha ,\beta ,\gamma \right)=\frac{\alpha \gamma \;\ln\;\left[\tan\left(\frac{\theta }{2}\right)\right]{\left[\tan\left(\frac{\theta }{2}\right)\right]}^{\beta }{\left[1+{\left\{\tan\left(\frac{\theta }{2}\right)\right\}}^{\beta }\right]}^{-\gamma -1}}{{\left[{\left\{1+{\left(\tan\left(\frac{\theta }{2}\right)\right)}^{\beta }\right\}}^{-\gamma }+\alpha -1\right]}^{2}}\text{,}$$

and

$$\Delta_{3}\left(y_{\left(i\right)}|\alpha ,\beta ,\gamma \right)=\frac{\alpha \;\ln\;\left[1+{\left\{\tan\left(\frac{\theta }{2}\right)\right\}}^{\beta }\right]{\left[1+{\left\{\tan\left(\frac{\theta }{2}\right)\right\}}^{\beta }\right]}^{\gamma }}{{\left[{\left\{1+{\left(\tan\left(\frac{\theta }{2}\right)\right)}^{\beta }\right\}}^{-\gamma }+\alpha -1\right]}^{2}}\text{.}$$

The weighted least squares (WLS) estimates ${\hat{\alpha }}_{WLS}$, ${\hat{\beta }}_{WLS}$ and ${\hat{\gamma }}_{WLS}$, are obtained by minimizing the expression given by

(34) $$\text{WLS}\left(\alpha ,\beta ,\gamma \right)=\sum_{i=1}^{n}\frac{{\left(n+1\right)}^{2}\left(n+2\right)}{i\left(n-i+1\right)}{\left[F\left(Y_{\left(i:n\right)}|\alpha ,\beta ,\gamma \right)-\frac{i}{\left(n+1\right)}\right]}^{2}\text{.}$$

Alternatively, Eq. (34) takes the form corresponding to Eq. (7), and is given by

(35) $$\text{WLS}\left(\alpha ,\beta ,\gamma \right)=\sum_{i=1}^{n}\frac{{\left(n+1\right)}^{2}\left(n+2\right)}{i\left(n-i+1\right)}{\left[\frac{1-{\left[1+{\left\{\tan\left(\frac{\theta }{2}\right)\right\}}^{\beta }\right]}^{-\gamma }}{1-\left(1-\alpha \right){\left[1+{\left\{\tan\left(\frac{\theta }{2}\right)\right\}}^{\beta }\right]}^{-\gamma }}-\frac{i}{\left(n+1\right)}\right]}^{2}\text{.}$$

The WLS estimates of the parameters α, β, and γ are obtained by taking the partial derivative of Eq. (35) with respect to each parameter. After some mathematical simplifications, the WLS estimates are given as follows

$$\sum_{i=1}^{n}\frac{{\left(n+1\right)}^{2}\left(n+2\right)}{i\left(n-i+1\right)}\left[\frac{1-{\left[1+{\left\{\tan\left(\frac{\theta }{2}\right)\right\}}^{\beta }\right]}^{-\gamma }}{1-\left(1-\alpha \right){\left[1+{\left\{\tan\left(\frac{\theta }{2}\right)\right\}}^{\beta }\right]}^{-\gamma }}-\frac{i}{\left(n+1\right)}\right]\Delta_{s}\left(y_{\left(i\right)}|\alpha ,\beta ,\gamma \right)=0,\;s=1,2,3$$

where, $\Delta_{s}\left(y_{\left(i\right)}|\alpha ,\beta ,\gamma \right)=0,\;\&\;s=1,2,3$ is defined above.

### 5.3 Cramér–von Mises estimation

Let $\theta_{1},\theta_{2},\ldots ,\theta_{n}$ be an ordered observation from sample from StSC-MOE Burr-XII random variables. The Cramér–Von Mises is used to estimate the parameters, say, ${\hat{\alpha }}_{CVM}$, ${\hat{\beta }}_{CVM}$, and ${\hat{\gamma }}_{CVM}$ which are determined by minimizing the function given by,

(36) $$\text{CVM}\left(\alpha ,\beta ,\gamma \right)=\frac{1}{12n}+\sum_{i=1}^{n}{\left[F\left(Y_{\left(i:n\right)}|\alpha ,\beta ,\gamma \right)-\frac{2i-1}{2n}\right]}^{2}\text{.}$$

Alternatively, Eq. (36) takes the form corresponding to Eq. (7), and is given by

(37) $$\text{CVM}\left(\alpha ,\beta ,\gamma \right)=\frac{1}{12n}+\sum_{i=1}^{n}{\left[\frac{1-{\left[1+{\left\{\tan\left(\frac{\theta }{2}\right)\right\}}^{\beta }\right]}^{-\gamma }}{1-\left(1-\alpha \right){\left[1+{\left\{\tan\left(\frac{\theta }{2}\right)\right\}}^{\beta }\right]}^{-\gamma }}-\frac{2i-1}{2n}\right]}^{2}\text{.}$$

Differentiate Eq. (37) with respect to α, β and γ, the estimates of the parameters can be determined numerically by the equations given below

$$\sum_{i=1}^{n}\left[\frac{1-{\left[1+{\left\{\tan\left(\frac{\theta }{2}\right)\right\}}^{\beta }\right]}^{-\gamma }}{1-\left(1-\alpha \right){\left[1+{\left\{\tan\left(\frac{\theta }{2}\right)\right\}}^{\beta }\right]}^{-\gamma }}-\frac{2i-1}{2n}\right]\Delta_{s}\left(y_{\left(i\right)}|\alpha ,\beta ,\gamma \right)=0,\;s=1,2,3$$

where $\Delta_{s}\left(y_{\left(i\right)}|\alpha ,\beta ,\gamma \right)$ are defined in section 5.2.

### 5.4 Anderson–Darling estimation

Let $\theta_{1},\theta_{2},\ldots ,\theta_{n}$ be ordered observations from a sample of StSC-MOE Burr-XII random variables. The Anderson–Darling used to find the parameters ${\hat{\alpha }}_{AD}$, ${\hat{\beta }}_{AD}$, and ${\hat{\gamma }}_{AD}$ that are determined by minimizing the function that are given below,

(38) $$A\left(\alpha ,\beta ,\gamma \right)=-n-\frac{1}{n}\;\sum_{i=1}^{n}\left(2i-1\right)\left\{logF\left(x_{1:n}|\alpha ,\beta ,\gamma \right)+\log\bar{F}\left(x_{n+1-i:n}|\alpha ,\beta ,\gamma \right)\right\}\text{.}$$

The non-linear equations given in Eq. (38), is defined corresponding to Eq (7), and given by

(39) $$A\left(\alpha ,\beta ,\gamma \right)=-n-\frac{1}{n}\;\sum_{i=1}^{n}\left(2i-1\right)\left[\begin{array}{c} \log\left\{\frac{1-{\left[1+{\left\{\tan\left(\frac{\theta }{2}\right)\right\}}^{\beta }\right]}^{-\gamma }}{1-\left(1-\alpha \right){\left[1+{\left\{\tan\left(\frac{\theta }{2}\right)\right\}}^{\beta }\right]}^{-\gamma }}\right\}+\\ \log\left\{1-\frac{1-{\left[1+{\left\{\tan\left(\frac{\theta }{2}\right)\right\}}^{\beta }\right]}^{-\gamma }}{1-\left(1-\alpha \right){\left[1+{\left\{\tan\left(\frac{\theta }{2}\right)\right\}}^{\beta }\right]}^{-\gamma }}\right\} \end{array}\right]$$

with respect to parameters α, β, and γ.

The non-linear equations given in Eq (39) is solved to obtain the estimates of α, β, and γ.

## 6 Simulation study to compare various estimation techniques

A simulation study serves as a statistical research approach utilized for assessing and contrasting various estimation methods. This is achieved by generating simulated datasets and employing different estimation techniques. The main goal is to assess the effectiveness of these methods across a range of scenarios and circumstances. By conducting the study within a controlled setting with predefined population parameters, direct comparisons can be made between the estimated values and the actual values. This comparative analysis enables the evaluation of accuracy, precision, bias, efficiency, and robustness of the estimation methods. In the field of statistical research, simulation studies provide important insights into the performance attributes of estimate techniques.

A comprehensive simulation study is discussed to observe and evaluate the behavior of the estimation techniques discussed in this section. The focus of the study is on parameter estimation of the StSC-MOE Burr-XII distribution. Numerical results obtained from the simulation study are presented to provide a detailed analysis of the findings. We conducted a simulation study by generating a total of 10 000 samples from the StSC-MOE Burr-XII distribution. The sample sizes used were n = 20, 50, 100, and 300. The parameter settings for the distribution were chosen as (α, β, γ) = {(1.7, 1.6, 1.2), (1, 3.5, 1), (4, 3, 4)}. The random number generation process was carried out using the QF as given by Eq. (10). We computed the empirical means, biases, standard errors, and mean square errors (MSEs) of all estimators to compare their performance across different sample sizes. We found that conducting 10 000 iterations provided stable and reliable results. The empirical bias and MSE were calculated using the given parameter settings (α, β, γ). Where the formulas for the bias and MSE is given below

$${Bias}_{\theta }=\sum_{i=1}^{N}\left({\hat{\theta }}_{i}-\theta \right)\text{,}$$

and

$${MSE}_{\theta }=\frac{1}{N}\sum_{i=1}^{N}{\left({\hat{\theta }}_{i}-\theta \right)}^{2}\text{.}$$

All the estimation outcomes and numerical findings are obtained using the software R Studio.

From Table 2, it can be observed that the MSE for all estimators are decreasing with the increase in sample sizes. For small sample sizes MLE performs best and then WLS, AD, CVM and OLS in the respective order to estimate the α, β, & γ, and for large sample sizes WLS performs best and WLS second best to estimate α, while MLE performs best and WLS second best estimator to estimate β, & γ.

**Table 2:** Parameter's Estimated values, Average Bias, Mean Standard Error, and Mean Relative Estimates for α=3, β=2.5, γ=3

Methods		α=3				β=2.5				γ=3			
		20	50	100	300	20	50	100	300	20	50	100	300
**MLE**	Average Bias	2.7241	2.9209	3.0480	3.1347	2.6133	2.5002	2.4427	2.3988	2.0337	2.1156	2.1655	2.1965
	Bias	0.4697	0.3003	0.2010	0.1438	0.3968	0.2705	0.1950	0.1351	0.9696	0.8846	0.8345	0.8035
	MSE	0.6104	0.2532	0.0939	0.0222	0.2117	0.1094	0.0579	0.0266	1.1019	0.8620	0.7353	0.6570
	MRE	0.1566	0.1001	0.0670	0.0479	0.1587	0.1082	0.0780	0.0540	0.3232	0.2949	0.2782	0.2678
**AD**	Average Bias	2.6622	2.8470	2.9895	3.1193	2.5091	2.4255	2.3648	2.3152	1.9402	2.0382	2.1039	2.1594
	Bias	0.5359	0.3709	0.2510	0.1530	0.4260	0.3072	0.2404	0.1986	1.0625	0.9619	0.8961	0.8407
	MSE	0.8233	0.4156	0.1811	0.0325	0.2420	0.1357	0.0840	0.0524	1.3561	1.0330	0.8551	0.7194
	MRE	0.1786	0.1236	0.0837	0.0510	0.1704	0.1229	0.0961	0.0794	0.3542	0.3206	0.2987	0.2802
**CVM**	Average Bias	2.5943	2.7319	2.8846	3.0778	2.5752	2.4578	2.3739	2.2884	1.8764	1.9536	2.0314	2.1202
	Bias	0.5950	0.4703	0.3386	0.1820	0.4476	0.3344	0.2675	0.2288	1.1270	1.0465	0.9686	0.8798
	MSE	1.0032	0.6304	0.3402	0.0699	0.2606	0.1590	0.1034	0.0680	1.5346	1.2344	1.0130	0.7920
	MRE	0.1983	0.1568	0.1129	0.0607	0.1791	0.1338	0.1070	0.0915	0.3757	0.3488	0.3229	0.2933
**OLS**	Average Bias	2.5890	2.7615	2.9072	3.0820	2.4329	2.3832	2.3391	2.2764	1.8526	1.9597	2.0351	2.1215
	Bias	0.6031	0.4476	0.3215	0.1786	0.4715	0.3499	0.2822	0.2374	1.1499	1.0404	0.9650	0.8786
	MSE	1.0611	0.6008	0.3212	0.0646	0.2934	0.1712	0.1124	0.0721	1.6187	1.2249	1.0072	0.7891
	MRE	0.2011	0.1492	0.1072	0.0595	0.1886	0.1399	0.1129	0.0950	0.3833	0.3468	0.3217	0.2929
**WLS**	Average Bias	2.6903	2.9082	3.0501	3.1377	2.4434	2.4267	2.3928	2.3829	1.9492	2.0702	2.1426	2.1843
	Bias	0.5133	0.3200	0.2036	0.1430	0.4451	0.3040	0.2247	0.1486	1.0535	0.9299	0.8575	0.8157
	MSE	0.7863	0.3181	0.1048	0.0216	0.2635	0.1323	0.0744	0.0321	1.3412	0.9617	0.7787	0.6777
	MRE	0.1711	0.1067	0.0679	0.0477	0.1781	0.1216	0.0899	0.0594	0.3512	0.3100	0.2858	0.2719

From Table 3, it is concluded that MLE performs best to estimate α, β, & γ, while CVM is second best to estimate α and AD is second best to estimate β, & γ. For large sample sizes MLE performs best to estimate α, β, & γ while the 2nd, 3rd, 4th and 5th order to estimate the α is WLS, AD, CVM and OLS; to estimate the β order is AD, CVM, WLS and OLS; and to estimate the γ order is WLS, AD, CVM and OLS.

**Table 3:** Parameter's Estimated values, Average Bias, Mean Standard Error, and Mean Relative Estimates for α = 1, β = 2, γ = 3.

Methods		α=1				β=2				γ=3			
		20	50	100	300	20	50	100	300	20	50	100	300
**MLE**	Average Bias	1.1058	1.1200	1.1729	1.1770	2.0802	1.9747	1.8986	1.8540	2.3242	2.4152	2.4871	2.5617
	Bias	0.6773	0.5490	0.4904	0.3387	0.4032	0.2713	0.2278	0.1785	0.7682	0.6659	0.5852	0.4768
	MSE	0.7273	0.4683	0.3761	0.1899	0.2552	0.1146	0.0734	0.0455	1.0699	0.7517	0.5664	0.3387
MRE	0.6773	0.5490	0.4904	0.3387	0.2016	0.1356	0.1139	0.0892	0.2561	0.2220	0.1951	0.1589	
**AD**	Average Bias	1.1342	1.0784	1.0602	1.0131	2.0092	1.9599	1.9035	1.8845	2.1178	2.1954	2.2703	2.2976
	Bias	0.8015	0.6458	0.5306	0.3716	0.4542	0.3264	0.2412	0.1809	0.9823	0.8894	0.7979	0.7303
	MSE	0.9383	0.6168	0.4103	0.2122	0.2957	0.1614	0.0864	0.0477	1.8629	1.4098	1.0264	0.7328
MRE	0.8015	0.6458	0.5306	0.3716	0.2271	0.1632	0.1206	0.0905	0.3274	0.2965	0.2660	0.2434	
**CVM**	Average Bias	1.0356	0.9569	1.0121	0.9247	2.1019	2.0310	1.9274	1.9057	1.9726	1.9510	2.1238	2.1411
	Bias	0.8107	0.6976	0.5897	0.4129	0.4802	0.3694	0.2804	0.1894	1.1241	1.1269	0.9398	0.8851
	MSE	0.9224	0.6855	0.4904	0.2435	0.3383	0.2054	0.1165	0.0533	2.3605	2.0932	1.4377	1.0619
MRE	0.8107	0.6976	0.5897	0.4129	0.2401	0.1847	0.1402	0.0947	0.3747	0.3756	0.3133	0.2950	
**OLS**	Average Bias	1.0966	1.0418	1.0163	0.9162	1.9835	1.9293	1.9015	1.8984	1.8513	2.0316	2.1110	2.1219
	Bias	0.8911	0.7054	0.5954	0.4167	0.4916	0.3541	0.2840	0.1942	1.2401	1.0541	0.9534	0.9045
	MSE	1.1108	0.7060	0.4994	0.2465	0.3449	0.1866	0.1183	0.0562	2.7878	1.9206	1.4527	1.0949
MRE	0.8911	0.7054	0.5954	0.4167	0.2458	0.1771	0.1420	0.0971	0.4134	0.3514	0.3178	0.3015	
**WLS**	Average Bias	1.1449	1.0892	1.0971	1.0843	1.9464	1.9209	1.8898	1.8516	2.0062	2.1753	2.3122	2.3991
	Bias	0.8502	0.6594	0.5245	0.3478	0.4659	0.3178	0.2576	0.1929	1.0911	0.9075	0.7525	0.6297
	MSE	1.0587	0.6467	0.4122	0.1956	0.3091	0.1528	0.0966	0.0530	2.2403	1.4084	0.9158	0.5479
MRE	0.8502	0.6594	0.5245	0.3478	0.2330	0.1589	0.1288	0.0964	0.3637	0.3025	0.2508	0.2099	

Overall, the MSE for MLE is comparatively less with other compared estimation methods for the proposed distribution.

## 7 Real-life applications

In this section the applications have been divided into two parts: in 7.1. Section the StSC-MOE Burr-XII distribution is applied on Posterior Corneal Curvature eye data because StSC-MOE Burr-XII is only suitable for the semi-circle data sets. In 7.2. Section the la-MOE Burr-XII distribution is suitable for circle and semi-circle both type data sets depend on the special cases.

### 7.1 Applications of StSC-MOE Burr-XII distribution

The StSC-MOE Burr-XII distribution's applications are illustrated in this section, along with a comparison of its performance with those of other existing distributions, such as hc-M Burr-III, hc-Burr-III, hc-GIW, hc-Log Logistic, hc-Gamma and hc-Burr-XII. The Akaike information criterion (AIC), Consistent Akaike Information Criterion, and Bayesian information criterion (BIC) are model selection methods used to find the best model. The model with the lowest test statistic is the one that works best for these selection processes.

**Fig. 4:**
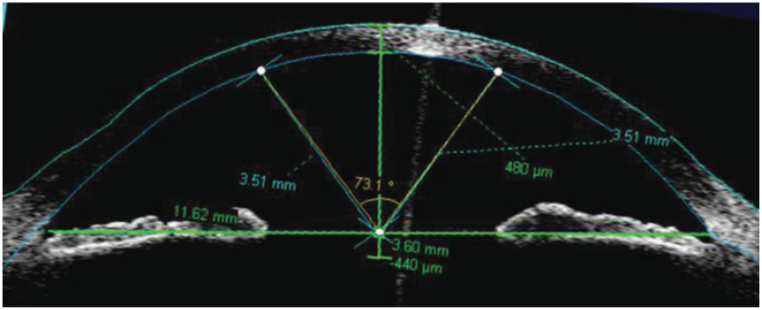
Measurement of the posterior corneal curvature.

Data explanation: Eye data of Posterior Corneal Curvature (Radian): The data acquired from the images of the posterior segment of the eyes of 23 patients and it used by Abuzaid [3]. Fig. 4 illustrates how the angle that measures the posterior corneal curvature is our variable of interest, which is a semicircle. An image of the posterior segment is shown in Fig. 4, where O is the point where the line drawn between the nasal and temporal scleral spurs (vertical line) and the geometrical axis of the eye (horizontal line) cross. The posterior corneal measurements (in radians) for 23 patients' eyes are as follows.

**Data-1 →** (posterior corneal curvature)**:** 1.60, 1.21, 1.46, 2.10, 1.40, 1.82, 1.57, 1.56, 1.85, 0.60, 1.70, 1.97, 1.47, 1.74, 1.67, 1.38, 0.53, 1.69, 1.63, 1.56, 1.81, 2.09, 2.29.

Table 4 includes the modeling of some semi-circular distributions including log-likelihoods, AICs, BICs, CAIDs, Ads, CVM(W) and KS statistics with p-values on semi-circular eye data. The StSC-MOE Burr-XII distribution shows more flexibility over the other competing semi-circular distributions due to the lowest AIC, BIC, CAID, AD, CVM, log-likelihood value, and larger p-value of the KS test.

**Table 4:** ML estimates, SE, Model Selection Criteria and Parameter Estimates for Eye data.

Model	ML estimates and SE			2l	AIC	BIC	CAIC	A	W	KS(p-value)
**StSC-MOE Burr-XII**	24.7067 (51.4809)	2.0786 (1.1269)	4.1830 (2.8076)	18.5834	24.5835	27.9900	25.8466	0.3721	0.0563	0.1040(0.9648)
**hc-MBIII**	2.6984 (2.1997)	6.6498 (2.0029)	6.2869 (7.6883)	19.2383	25.2383	28.6447	26.5014	0.4452	0.0678	0.1274(0.8497)
**hc-Burr-III**	1.0005 (0.2295)	4.2867 (0.8574)	–	22.7448	26.7448	29.0158	27.3448	0.8152	0.1191	0.1839(0.4180)
**hc-GIW**	0.82339 (52.2104)	1.7021 (0.2367)	0.9109 (98.3115)	37.5461	43.5461	46.9526	44.8092	2.4295	0.4125	0.2732(0.0645)
**hc-Log Logistic**	1.0643 (0.0844)	4.3849 (0.8006)	–	22.1443	26.1442	28.4152	26.7442	0.7408	0.1075	0.1165(0.9136)
**hc-Gamma**	5.7177 (1.6383)	0.1933 (0.0579)	–	22.1746	26.1746	28.4456	26.7746	0.8167	0.1272	0.1699(0.5203)
**hc-BIII**	4.3794 (0.8908)	0.9475 (0.2206)	–	22.6901	26.6901	28.9611	27.2901	0.8186	0.1195	0.1655(0.5544)

### 7.2 Applications of *la*-MOE Burr-XII distribution (Case-I: C-MOE Burr-XII)

This section demonstrates the applications of the *la*-MOE Burr-XII distribution on circular data sets and compares its performance to that of other competing distributions. The Akaike information criterion (AIC), Consistent Akaike Information Criterion, and Bayesian information criterion (BIC) are model selection methods used to find the best model. The model with the lowest test statistic is the one that works best for these selection processes. The *la*-MOE Burr-XII distribution case-I which is C-MOE Burr-XII distribution is applied on two circular data sets.

**Data-2 →** (Wind direction data): The dataset contains 21 wind directions by a Milwaukee weather station, at 6.00 a.m. on consecutive days.

356 97 211 232 343 292 157 302 335 302 324 85 324 340 157 238 254 146 232 122 329.

**Data-3 →** (Vanishing angles of homing pigeons): This dataset includes the vanishing angles of 13 homing pigeons that were released individually in the sub-Alpine Toggenburg Valley of Switzerland.

20 135 145 165 170 200 300 325 335 350 350 350 355.

**Fig. 5:**
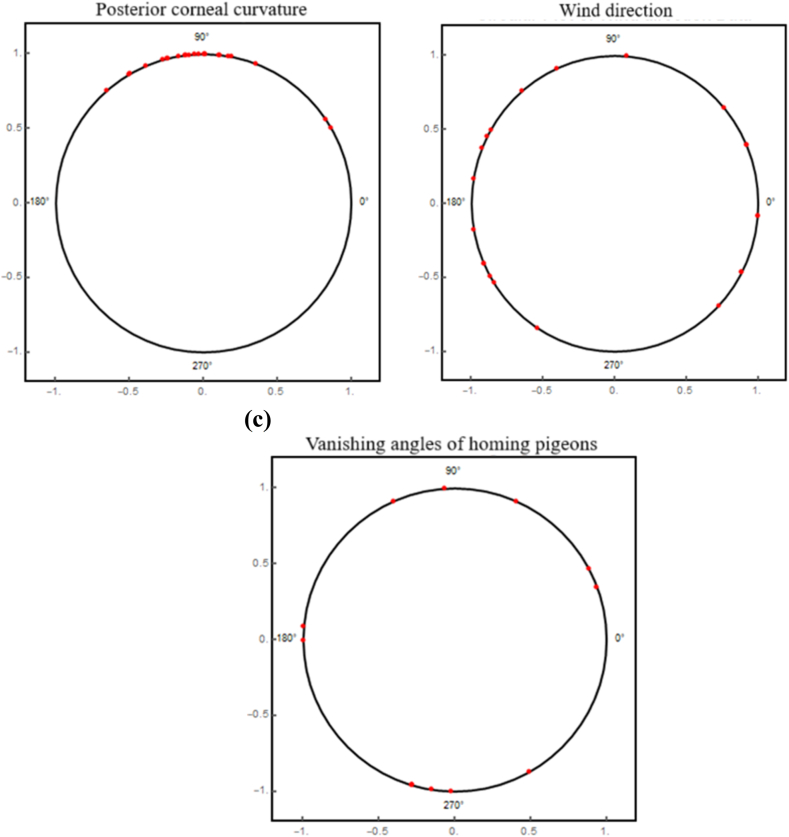
(a) Semi-circular plot for the Posterior corneal curvature, (b) Circular plot for the Wind direction, (c) Circular plot for the Vanishing angles of homing pigeons.

Fig. 5 exhibits the representation of the semi-circular eye data, and circular representations of the wind direction data, and the vanishing angles of homing pigeons.

The proposed model's applications are approached through two methods: (i) the StSC-MOE Burr-XII distribution and (ii) the *la*-MOE Burr-XII distribution (Case I). Upon analyzing the Eye posterior corneal curvature dataset (semi-circle data), the StSC-MOE Burr-XII distribution emerges as the best fit due to its attainment of the lowest values for metrics such as AIC, BIC, CAIC, AD, W, log likelihood, and the highest p-value for the Kolmogorov-Smirnov goodness-of-fit test. Subsequently, the *la*-MOE Burr-XII distribution is selectively applied: Case I (C-MOE Burr-XII), tailored for circular datasets, is employed on a few circular datasets, while Case II aligns with the StSC-MOE Burr-XII distribution. Additionally, Case IV (SC Burr-XII) caters to semi-circular data, making it suitable for eye data analysis. Overall, the *la*-MOE distribution also exhibits greater flexibility in accommodating both circular and semi-circular datasets compared to competing distributions.

Table 5, Table 6 include the modeling of some circular distributions including log-likelihoods, AICs, BICs, CAIDs, Ads, CVM(W) and KS statistics with p-values on two circular data sets wind direction, and homing pigeons. The *la*-MOE (C-MOE) Burr-XII distribution is more flexible as compared to the other competing circular distributions due to the lowest AIC, BIC, CAID, AD, CVM, log-likelihood value, and larger p-value of the KS test.

**Table 5:** ML estimates, SE, Model Selection Criteria and Parameter Estimates for Wind direction.

Model	ML estimates and SE			2l	AIC	BIC	CAIC	A	W	KS(p-value)
**C-MOE Burr-XII**	1.5744 (1.5242)	1.9089 (0.7509)	0.5328 (0.3826)	33.5809	73.1618	76.2954	74.5736	0.2895	0.0454	0.1175(0.9338)
**C-GLI**	2.1265 (0.5324)	0.7619 (0.1532)	–	36.9678	77.9356	80.0246	78.6022	28.1044	4.6267	0.7954(0.0000)
**C-GLII**	0.5445 (0.1269)	1.2259 (0.2437)	–	36.6704	77.3408	79.4298	78.0075	28.0691	4.6615	0.7920(0.0000)
**C-SGL**	1.1112 (0.2106)	−0.1007 (0.4121)	–	37.8322	79.6644	81.7535	80.3311	29.2152	4.7582	0.8112(0.0000)
**C-WL**	12.5349 (36.5184)	32.4381 (93.8138)	–	34.1129	72.2258	74.3148	72.8924	0.6423	0.0975	0.9766(0.0000)
**C-WE**	−0.3863 (0.1378)	–	–	34.0929	70.1859	71.2304	70.3964	0.3112	0.0485	0.1035(0.9781)

**Table 6:** ML estimates, SE, Model Selection Criteria and Parameter Estimates for Homing pigeon.

Model	ML estimates and SE			2l	AIC	BIC	CAIC	A	W	KS(p-value)
**C-MOE Burr-XII**	10.8036 (23.0822)	1.4019 (0.7605)	5.8904 (3.0460)	17.1162	40.2324	41.9272	42.8991	0.8538	0.1375	0.2067 (0.6353)
**C-GLI**	2.9719 (0.8628)	0.3896 (0.0951)	–	27.1100	58.2199	59.3498	59.4199	17.5371	2.8430	0.7146(0.0000)
**C-GLII**	−10.2974 (0.1234)	−6.4040 (0.1234)	–	−473.904	943.8077	942.6778	942.6077	–	–	1.0000(0.0000)
**C-SGL**	2.0388 (0.5059)	0.8278 (0.9448)	–	27.6825	59.3650	60.4949	60.5650	18.1203	2.9358	0.7343(0.0000)
**C-WL**	1.4292 (0.6553)	3.1833 (1.3815)	–	21.1088	46.2175	47.3474	47.4175	0.8032	0.1324	0.7596(0.0000)
**C-WE**	−0.3836 (0.1748)	–	–	21.1416	44.2833	44.8482	44.6469	0.5661	0.0916	0.2365(0.4610)

**Fig. 6:**
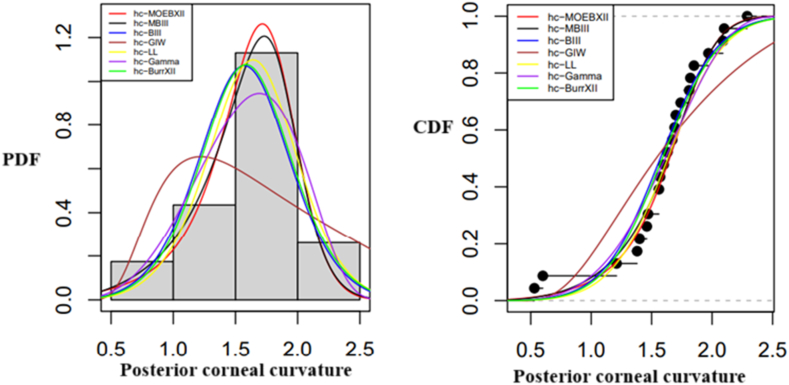
(a) Fitted and empirical pdf's of the Posterior corneal curvature, (b) Fitted and empirical cdf's of the Posterior corneal curvature (StSC-MOE Burr-XII distribution).

**Fig. 7:**
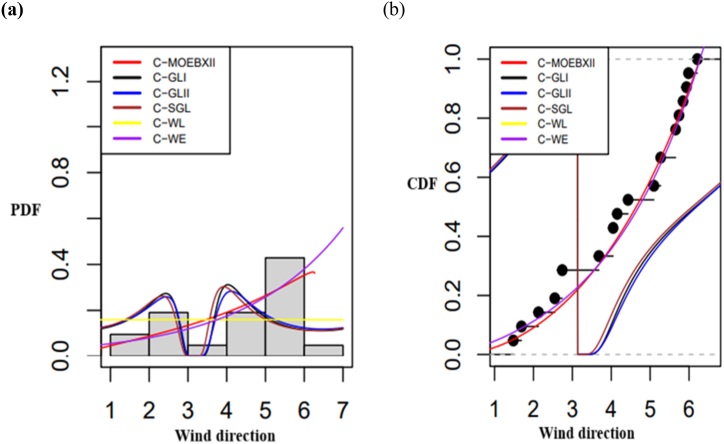
(a) Fitted and empirical pdf's of Wind direction, (b) Fitted and empirical cdf's of the Wind direction la-MOE (C-MOE Burr-XII distribution).

**Fig. 8:**
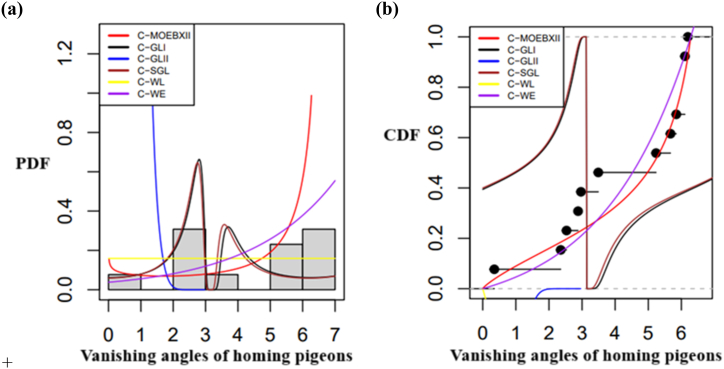
(a) Fitted and empirical pdf's, of the Vanishing angles of homing pigeons, (b) Fitted and empirical cdf's of the Vanishing angles of homing pigeons (la-MOE (C-MOE) Burr-XII distribution).

In Fig. 6a and b, the pdfs and cdfs of various distributions fitted to the eye dataset are shown, respectively. Fig. 6a compares the fitted pdfs with empirical pdfs, whereas Fig. 6b compares the fitted cdfs with empirical cdfs, helping to assess how well the distributions describe the data. The graph shows that, in comparison to other models, the StSC-MOE Burr-XII distribution has the best fit for the dataset. Similarly, Fig. 7(a and b), and 8(a, b) illustrate the fitted and empirical pdf's and cdf's of several distributions that are used to describe the wind direction and homing pigeon's datasets. Fig. 7(a and b) and 8(a, b) show that, in comparison to other models, the *la*-MOE (C-MOE) Burr-XII distribution has the best fit for the dataset.

**Fig. 9:**
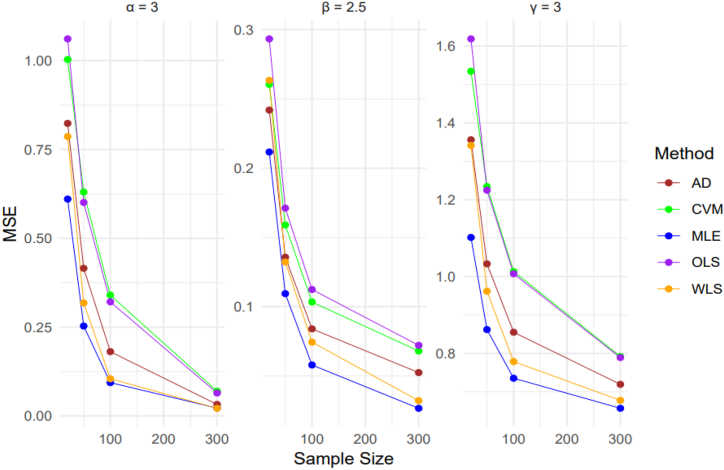
MSE of the parameter estimates with different estimation methods and sample sizes for Table 2.

**Fig. 10:**
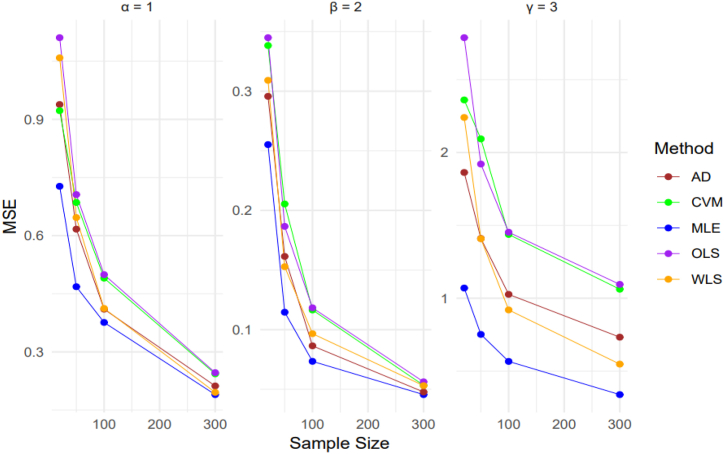
MSE of the parameter estimates with different estimation methods and sample sizes for Table 3.

Fig. 9, Fig. 10 illustrate that as the sample size increases, the MSE decreases for all five estimation methods. Overall, MLE performs better compared to AD, CVM, OLS, and WLS across all three parameters of the proposed distribution, for all sample sizes. Similarly, Table 2, Table 3 show that as the sample size increases, the MSE for all five estimation methods decreases, with MLE consistently having a lower MSE than AD, CVM, OLS, and WLS. Generally, it is concluded that MLE outperforms the other methods for small, medium, and large samples when estimating the proposed density. Based on these findings, MLE is recommended for estimating the proposed density in real-life data sets.

### 7.3 Discussion

The considerable potential in modeling circular and semi-circular datasets is highlighted by the comparative study of the StSC-MOE Burr-XII and *la*-MOE Burr-XII distributions. With the lowest values for AIC, BIC, CAIC, AD, W, and log-likelihood, as well as the greatest p-value for the Kolmogorov-Smirnov goodness-of-fit test, the StSC-MOE Burr-XII distribution was found to be the most appropriate model for the Eye posterior corneal curvature dataset. All of these metrics confirm that it is accurate and resilient in capturing the subtleties of semi-circular data.

Furthermore, circular datasets like wind direction and homing pigeons showed better performance when the la-MOE Burr-XII distribution—more specifically, the C-MOE Burr-XII (Case I)—was used. These results are corroborated by a stronger p-value for the KS test and lower values for AIC, BIC, CAID, AD, CVM, and log-likelihood. By using their fitted and empirical pdfs and cdfs, Fig. 6, Fig. 7, Fig. 8 graphically show the superior fit of the StSC-MOE Burr-XII and *la*-MOE (C-MOE) Burr-XII distributions over other models.

A larger sample size results in a lower Mean Squared Error (MSE) for each of the five estimation techniques—Maximum Likelihood Estimation (MLE), Anderson-Darling (AD), Cramér-von Mises (CVM), Ordinary Least Squares (OLS), and Weighted Least Squares (WLS)—as shown in Fig. 9, Fig. 10 and supported by the data in Table 2, Table 3 MLE consistently showed the lowest MSE among them, demonstrating its effectiveness in parameter estimation for the suggested distributions regardless of sample size. This steady performance highlights MLE's dependability as the go-to estimating technique for real-world data processing.

In summary, the *la*-MOE Burr-XII distribution provides significant flexibility for both circular and semi-circular data, while the StSC-MOE Burr-XII distribution is quite effective for semi-circular datasets. It is recommended that MLE be used for estimating the proposed densities in real-world datasets due to its constant superiority in minimizing MSE across a range of sample sizes. This thorough examination highlights how important it is to choose the right distribution models and estimating methods in order to improve the precision and dependability of statistical conclusions in a range of applications.

## 8 Conclusion

This study introduces a novel semi-circular distribution by employing an inverse stereographic projection (ISP) technique on the density function of the MOE Burr-XII distribution. Various characteristics of the proposed distribution have been derived. Additionally, several reliability measures for the StSC-MOE Burr-XII distribution have been established. The study presents linear and polar representations of the density function and hazard plots. The density and hazard plots of the proposed distribution, illustrate increasing, decreasing, and bathtub-shaped forms, indicating the flexibility of the proposed distribution in accommodating various shapes of datasets. Then, a stereographic *l*-axial distribution is introduced for the StSC-MOE Burr-XII distribution, termed as the *la*-MOE Burr-XII distribution.

This study identifies five estimation methods for the proposed StSC-MOE Burr-XII distribution, followed by a simulation study aimed at evaluating the performance of these estimation techniques. Based on the simulation study, it is concluded that MLE outperforms AD, CVM, OLS, and WLS across all three parameters of the proposed distribution, for all sample sizes. MLE is therefore recommended for estimating the proposed density for small, medium, and large samples. These findings suggest that MLE should be used for estimating the proposed density in real-life data sets.

However, our primary focus is on introducing the semi-circular MOE Burr-XII distribution, tailored for datasets where observations are in radians within the (0,π) range. This proposed density model is specifically designed for semi-circular data, such as the posterior corneal curvature of the eye, and demonstrates favorable outcomes compared to alternative models. The proposed semi-circular model holds significant potential for applications in the medical field, aiding in decision-making regarding treatments and medications. Subsequently, the proposed model is extended to the *la*-MOE Burr-XII distribution, which can be adapted for both circular and semi-circular applications depending on specific cases within the *la*-MOE Burr-XII distribution framework. For semi-circular datasets, the model is suitable for eye data analysis, while for circular datasets, such as wind direction and homing pigeon movements, it offers applicable solutions.

Overall, this research stands out for its applicability to highly sensitive semi-circular datasets, such as corneal curvature data, as well as directional datasets like wind direction and homing pigeon movements, which typically require specialized directional distributions for accurate modeling.

## 9 Recommendations

Based on the extensive analysis presented in this study, the following recommendations are made for future research and practical applications.

• For modeling semi-circular data, the StSC-MOE Burr-XII distribution has proven to be remarkably versatile and flexible. It is advised to assess the StSC-MOE Burr-XII distribution's performance in various scenarios by contrasting it with other circular and semi-circular models already in use. The best model for a given collection of data may be chosen with the use of goodness-of-fit tests and information criteria such as AIC, BIC, CAIC, HQIC, and KS test with p-value.
• Five parameter estimate techniques are evaluated in the paper: MLE, AD, CVM, OLS, and WLS. Due to its efficiency and consistency, MLE is usually advised, however CVME and ADE have also demonstrated their ability to produce reliable estimates for large sample sizes.
• Applications where semi-circular data is frequently encountered, such environmental sciences, medical research, and meteorology, are especially well-suited for the StSC-MOE Burr-XII distribution. To take use of this model's potential advantages and add to the growing body of knowledge on circular data analysis, practitioners are urged to incorporate it into their analyses.
• The creation of Bayesian estimation techniques for the StSC-MOE Burr-XII distribution may be the subject of future investigations. Furthermore, expanding the model to manage multivariate semi-circular data can open up fresh avenues for investigation and useful applications.

## 10 Patents

NA.

## CRediT authorship contribution statement

**Shakila Bashir:** Writing – review & editing, Writing – original draft, Validation, Supervision, Software, Resources, Project administration, Methodology, Investigation, Formal analysis, Data curation, Conceptualization. **Bushra Masood:** Writing – original draft, Validation, Software, Methodology, Formal analysis, Data curation, Conceptualization. **Aamir Sanaullah:** Writing – review & editing, Validation, Supervision, Resources, Project administration, Investigation, Data curation. **Laila A. Al-Essa:** Writing – review & editing, Resources, Project administration, Investigation, Funding acquisition, Validation.

## Ethical statement

There are no human/animal subjects in this article therefore an ethics statement is not applicable because this study is applied on already published data.

## Data availability statement

Data included in article/supp. Material/referenced in article.

## Use of AI tools declaration

The authors declare that they have used Chat GPT as an artificial intelligence tool only to improve the readability of this article. After using this tool, the author(s) reviewed and edited the content as needed and take(s) full responsibility for the content of the publication.

## Funding

The APC was funded by Princess Nourah bint Abdulrahman University, Riyadh, Saudi Arabia, under the grant number, PNURSP2025R443.

## Declaration of competing interest

The authors declare that they have no known competing financial interests or personal relationships that could have appeared to influence the work reported in this paper.
